# Supplementary material for: Association Between Circulating Vitamin K Levels, Gut Microbiome, and Type 1 Diabetes: A Mendelian Randomization Study
Source: Nutrients. 2024 Nov 5;16(22):3795. doi: 10.3390/nu16223795 (PMC11597649; doi:10.3390/nu16223795)
Supplement: Supplementary file 1 [file nutrients-16-03795-s001.zip › MR-STROBE_checklist_VitK_T1D.pdf]

Supplementary Table S11: MR-STROBE checklist.

Item No.	Section	Checklist item	Page No.	Relevant text from manuscript
1	**TITLE and ABSTRACT**	Indicate Mendelian randomization (MR) as the study's design in the title and/or the abstract if that is a main purpose of the study	1	A Mendelian Randomization Study was applied to test whether genetically vitamin K (VK) levels are causally associated with the risk of type 1 diabetes (T1D).
	**INTRODUCTION**			
2	**Background**	Explain the scientific background and rationale for the reported study. What is the exposure? Is a potential causal relationship between exposure and outcome plausible? Justify why MR is a helpful method to address the study question	2-3	While vitamin K (VK) has historically been recognized for its role in blood coagulation and as an antioxidant and anti-inflammatory agent, recent studies suggest its potential involvement in glucose homeostasis [1]. Collectively, the evidence from the aforementioned epidemiological studies faces challenges in establishing causal associations between VK and T1D due to inherent limitations, such as unmeasured confounding and reverse causation[1, 3-5, 9, 10, 13, 16-19]. Mendelian randomization (MR) has emerged as a powerful tool leveraging genetic variants associated with biomarkers to investigate causal

				relationships of modifiable exposures with disease outcomes. Unlike observational studies, MR minimizes biases from confounding and reverse causation by using germline genetic variants randomly assigned at conception[20-28]. MR has been used previously to investigate the causal role of various biomarkers in T1D[23, 26].
3	**Objectives**	State specific objectives clearly, including pre-specified causal hypotheses (if any). State that MR is a method that, under specific assumptions, intends to estimate causal effects	3	In this study, we aimed to test whether circulating VK1 levels are causally associated with the risk of T1D using two-sample MR. Given the role of the gut microbiome in VK2 synthesis and T1D and the tight homeostasis between VK1 and VK2, we studied the potential effect of specific gut microbiome populations on the association between VK and T1D using multivariable MR.
	**METHODS**			
4	**Study design and data sources**	Present key elements of the study design early in the article. Consider including a table listing sources of data for all phases of the study. For each data source contributing to the analysis, describe the following:	3-4	See Figure 1 & 2
	a)	Setting: Describe the study design and the underlying population, if possible. Describe	3-4	We performed this study using a conventional two-sample MR design,

	the setting, locations, and relevant dates, including periods of recruitment, exposure, follow-up, and data collection, when available.		assessing the association between T1D and vitamin K, and Figure 1 provides a summary of the design that is being used for the current investigation. Firstly, we extracted available genetic IVs from the T1D European analysis. Secondly, the summary data comprising all SNPs from the only available GWAS for VK levels were collected. To evaluate the causal effect, we employed univariate two-sample MR and various sensitivity analyses. In addition, we conducted an MVMR analysis controlling for confounding variables, given the role of the gut microbiome in VK2 synthesis and T1D.
b)	Participants: Give the eligibility criteria, and the sources and methods of selection of participants. Report the sample size, and whether any power or sample size calculations were carried out prior to the main analysis	3-5	Sample size: T1D: 18,942, VK levels: 2,138<br>See Supplementary table 1
c)	Describe measurement, quality control and selection of genetic variants	4-6	We retrieved effects of SNPs associated with VK1 levels on T1D from the largest European T1D GWAS by Chiou et al. [31]. Through the meta-analysis, they combined the association results for 18,942 patients with European ancestry and 520,580 controls from 9 cohorts, that reached genome-wide significance ($P < 5 \times 10^{-8}$, linkage disequilibrium (LD); $r2 < 0.7$, LD distance > 10,000 kb). F-

				statistic >10 indicates a significant association between selected instrumental variables and T1D.
	d)	For each exposure, outcome, and other relevant variables, describe methods of assessment and diagnostic criteria for diseases	4-6	T1D: T1D diagnosis, insulin treatment within a year of diagnosis, no T2D diagnosis.<br>VK: Diagnosis was confirmed in all VK patients by direct review of gut microbiome reports and clinical VK1 and VK2 records.<br>Adjusted variables: These traits are detailed in previous studies.
	e)	Provide details of ethics committee approval and participant informed consent, if relevant		Not applied.
5	**Assumptions**	Explicitly state the three core IV assumptions for the main analysis (relevance, independence and exclusion restriction) as well assumptions for any additional or sensitivity analysis	4	There are three important assumptions of conventional MR analysis. The ideal IVs must satisfy as the following: (i) must be truly associated with T1D (in this study, defined as the genetic association with P <5×10-8); (ii) not associated with confounders of T1D and VK; (iii) should only be associated with the VK through T1D.
6	**Statistical methods: main analysis**	Describe statistical methods and statistics used		

	a)	Describe how quantitative variables were handled in the analyses (i.e., scale, units, model)	3-6	Using MR methods and OR
	b)	Describe how genetic variants were handled in the analyses and, if applicable, how their weights were selected	4-6	1. SNPs must reach genome-wide significance $P < 5\times10^{-8}$<br>2. Clumping technique<br>3. Harmonise
	c)	Describe the MR estimator (e.g. two-stage least squares, Wald ratio) and related statistics. Detail the included covariates and, in case of two-sample MR, whether the same covariate set was used for adjustment in the two samples	4-6	Wald ratio，Cochran's Q statistic<br>Covariates: See Supplementary Table 9. The same covariate set was used for adjustment in the two samples
	d)	Explain how missing data were addressed	3,5	No
	e)	If applicable, indicate how multiple testing was addressed		No
7	**Assessment of assumptions**	Describe any methods or prior knowledge used to assess the assumptions or justify their validity	4-5	IVW, MR-Egger, weighted median, and maximum likelihood
8	**Sensitivity analyses and additional analyses**	Describe any sensitivity analyses or additional analyses performed (e.g. comparison of effect estimates from different approaches, independent replication, bias analytic techniques, validation of instruments, simulations)	4-5	We used first-order IVWs and MR-Egger to generate Cochran's Q statistic to check for heterogeneity, which represents a possible violation of modelling assumptions. This study used the MR-Egger regression intercept examination to estimate the potential pleiotropy between exposure and outcome. A p value of <

				0.05 represented the existence of pleiotropy. Once heterogeneity or horizontal pleiotropy was noteworthy, we used MR-Pleiotropy Residual Sum and Outlier (MR-PRESSO) to remove outlier SNPs.
9	**Software and pre-registration**			
	a)	Name statistical software and package(s), including version and settings used	4-6	All analyses were carried out with the packages “TwoSampleMR”, “MR-PRESSO”, and “MVMR” of R (version 4.3.1).
	b)	State whether the study protocol and details were pre-registered (as well as when and where)		Not applicable
	**RESULTS**			
10	**Descriptive data**			
	a)	Report the numbers of individuals at each stage of included studies and reasons for exclusion. Consider use of a flow diagram	10	Supplementary Table 1 presents the characteristics of populations included in GWAS data on exposure and outcome.
	b)	Report summary statistics for phenotypic exposure(s), outcome(s), and other relevant variables (e.g. means, SDs, proportions)	10	These traits are detailed in previous studies.

	c)	If the data sources include meta-analyses of previous studies, provide the assessments of heterogeneity across these studies		We compiled individual-level genotype data and summary statistics for T1D from 18,942 patients with European ancestry and 520,580 controls from 9 cohorts. This is from the largest T1D GWAS by Chiou et al. For control cohorts, we also used phenotype files (where available) to remove individuals with other autoimmune diseases.
	d)	For two-sample MR:<br>i. Provide justification of the similarity of the genetic variant-exposure associations between the exposure and outcome samples<br>ii. Provide information on the number of individuals who overlap between the exposure and outcome studies	10	To our knowledge, the maximum sample overlapping rate between exposure data and outcome data is less than 1%
11	**Main results**			
	a)	Report the associations between genetic variant and exposure, and between genetic variant and outcome, preferably on an interpretable scale	11	Using 6 independent IVs and their effects on the exposure (VK1 levels) from each of the three models correcting for different covariates. In all three models, all 6 SNPs have an F-statistic above 10.
	b)	Report MR estimates of the relationship between exposure and outcome, and the measures of uncertainty from the MR analysis, on an interpretable scale, such as odds ratio or relative risk per SD difference	11	In the main univariable analyses, we identified a significant causal relationship between exposure and outcome ($P < 0.05$ across four MR methods), which referred to a causal association between T1D and increased VK level risk (OR: 0.933, 95%

				CI: 0.837 to 1.039 for IVW; OR: 0.984, 95% CI: 0.883 to 1.097 for weighted median and OR: 1.026, 95% CI: 0.698 to 1.507 for maximum likelihood)
	c)	If relevant, consider translating estimates of relative risk into absolute risk for a meaningful time period		No
	d)	Consider plots to visualize results (e.g. forest plot, scatterplot of associations between genetic variants and outcome versus between genetic variants and exposure)	11	Four methods were used to evaluate the results of MR analysis, and the scatter plot was generated (Figure 2 and Supplementary Tables S2-S6). Supplementary Tables 2-4 illustrated a relatively symmetrical distribution of variant effects for VK, indicating an absence of directional pleiotropy.
12	**Assessment of assumptions**			
	a)	Report the assessment of the validity of the assumptions	11-12	Additionally, we conducted several sensitivity analyses to determine potential heterogeneity and horizontal pleiotropy (Supplementary table 6).
	b)	Report any additional statistics (e.g., assessments of heterogeneity across genetic variants, such as I^2, Q statistic or E-value)	6	Neither Cochran's Q-test nor the MR Egger regression analysis detected heterogeneity and horizontal pleiotropy in the primary analysis (MR-Egger Ph = 0.08, IVW Ph = 0.14).

13	**Sensitivity analyses and additional analyses**			
	a)	Report any sensitivity analyses to assess the robustness of the main results to violations of the assumptions	11-12	No
	b)	Report results from other sensitivity analyses or additional analyses	6	Moreover, the Phenoscanner results are similar to those of the main analysis. From the MR, the results for mediating effects of gut microbiome were equally non-significant. (Supplementary table 5)
	c)	Report any assessment of direction of causal relationship (e.g., bidirectional MR)		No
	d)	When relevant, report and compare with estimates from non-MR analyses		No
	e)	Consider additional plots to visualize results (e.g., leave-one-out analyses)		No
	**DISCUSSIO N**			
14	**Key results**	Summarize key results with reference to study objectives	12-13	The two-sample MR analysis provided a null result. The MVMR analysis provided suggestive results.
15	**Limitations**	Discuss limitations of the study, taking into account the validity of the IV assumptions, other sources of potential bias, and	13-14	The studied population only includes individuals of European ancestry. Low statistical power was observed due to the

		imprecision. Discuss both direction and magnitude of any potential bias and any efforts to address them		presence of a small VK1 GWAS. Due to the lack of summary statistics from the VK1 GWAS, we were not able to exclude the possibility of the gut microbiome being a collider. The other two statistical models could introduce colliders which is a possible explanation for the observed null result in the MVMR.<br>The lack of an available VK2 GWAS led to the use of the gut microbiome as an informed mediator.<br>The traditional MR approach cannot assess non-linear effects, preventing us from evaluating whether only extreme levels of VK1 could influence T1D.
16	**Interpretation**			
	a)	Meaning: Give a cautious overall interpretation of results in the context of their limitations and in comparison with other studies	14	This MR study does not support the use of vitamin K supplements to prevent T1D.
	b)	Mechanism: Discuss underlying biological mechanisms that could drive a potential causal relationship between the investigated exposure and the outcome, and whether the gene-environment equivalence assumption is reasonable. Use causal language carefully,	14	The biological mechanisms remained unclear. Further investigation is needed to uncover the gut microbiome's relationship in the association between VK and T1D. Previous animal studies have excluded the gut microbiome as the main mediator in the conversion of VK1

		clarifying that IV estimates may provide causal effects only under certain assumptions		to VK2. However, some variables in the suggested mechanism of the conversion suggest that the gut microbiome might be implicated.
	c)	Clinical relevance: Discuss whether the results have clinical or public policy relevance, and to what extent they inform effect sizes of possible interventions	14	MR analysis indicated no effect of VK1 on T1D. However, the absence of evidence for a large causal effect of VK1 and VK2 on the risk of T1D can inform decision-making processes, such as conducting clinical trials or interventions.
17	**Generalizability**	Discuss the generalizability of the study results (a) to other populations, (b) across other exposure periods/timings, and (c) across other levels of exposure		NO
	**OTHER INFORMATION**			
18	**Funding**	Describe sources of funding and the role of funders in the present study and, if applicable, sources of funding for the databases and original study or studies on which the present study is based	15	
19	**Data and data sharing**	Provide the data used to perform all analyses or report where and how the data can be accessed, and reference these sources in the article. Provide the statistical code needed to reproduce the results in the article, or report	15	R scripts used to generate the results of this study are available upon request to the corresponding author. Summary-level results of all GWAS used in this study, except for the VK1 GWAS, are publicly available through the GWAS catalogue.

		whether the code is publicly accessible and if so, where		
20	**Conflicts of Interest**	All authors should declare all potential conflicts of interest	15	The authors declare no conflict of interest**.**
